# A case of rectal ulcers during aspirin therapy in acute Kawasaki disease

**DOI:** 10.1186/s12969-020-0414-6

**Published:** 2020-03-17

**Authors:** Yong Han, Chenmin Hu, Yanping Yu

**Affiliations:** Department of Pediatrics, Affiliated Hangzhou First People’s Hospital, Zhejiang University School of Medicine, Hangzhou, China

**Keywords:** Kawasaki disease, Gastrointestinal hemorrhage, Rectal ulcer, Aspirin

## Abstract

Kawasaki disease (KD) is an acute febrile multisystem vasculitis and has been recognized to be one of the most common causes of acquired heart disease in children. Although gastrointestinal symptoms including vomiting, diarrhea, and abdominal pain are not uncommon in KD patients, KD with lower gastrointestinal bleeding is quite rare. Here, we describe a 3-year-old boy with typical KD who had lower gastrointestinal bleeding caused by rectal ulcers on the third day of aspirin therapy.

Dear Editor,

Kawasaki disease (KD) is a febrile multisystem vasculitis of unknown etiology, especially involving coronary arteries. Although the prevalence of gastrointestinal involvement in KD is unknown, gastrointestinal symptoms including vomiting, diarrhea, and abdominal pain are relatively common. However, KD with lower gastrointestinal bleeding is quite rare. Here, we describe a 3-year-old boy with typical KD who had lower gastrointestinal bleeding caused by rectal ulcers on the third day of aspirin therapy.

A 3-year-old boy was first seen on day 2 of high fever. On physical examination, enlarged tonsil, pharyngeal hyperemia, and coarse respiratory sounds of both lungs were noted. No other abnormal finding was present at that time. Laboratory findings were as follows: white blood cell count 14.8*10^9/L, neutrophil 84.8%, hemoglobin 131 g/L, platelet count 228*10^9/L, C-reactive protein 122 mg/L. He was admitted and treated with intravenous antibiotics empirically. The boy remained febrile. Three day later (day 5), the onset of maculopapular rash on the trunk and limbs, nonexudative conjunctivitis, strawberry tongue, edema of hands and feet, and an enlarged right cervical lymph node led to the diagnosis of KD. Fortunately, echocardiography (Fig. 1A1, A2 )revealed no dilatation of coronary arteries (2.2 mm left and 2.0 mm right). Then, intravenous immunoglobulin (IVIG, 2 g/kg) and oral aspirin (50 mg/kg per day) were administered. After IVIG, his fever resolved rapidly. However, hematochezia occurred on day 3 of aspirin. Repeated laboratory examinations revealed white blood cell count of 7.1 × 10^9/L with predominance of neutrophil 51.2%, hemoglobin level of 118 g/L, platelet count of 378 × 10^9/L, and C-reactive protein of 57 mg/L. Colonoscopy revealed multiple superficial active ulcers in the rectum (Fig. 1B). Microscopic examination of a specimen from rectal biopsy revealed focal active colitis. There was predominantly neutrophilic infiltration within the lamina propria along with hemorrhage and crypt abscess formation (Fig. 1C). Thus, high-dose aspirin was stopped and intravenous methylprednisolone (30 mg per day) was given. Three days later, bloody stool disappeared. The intravenous methylprednisolone was changed to methylprednisolone tablets with gradually decreased dosage. Simultaneously, low-dose aspirin (4 mg/kg per day) was started. On day 15, most of the laboratory parameters were normal except for elevated platelet count of 623 × 10^9/L. The next day, he was discharged and continued with low-dose aspirin (4 mg/kg per day) for 8 weeks. Further follow-up showed that the patient recovered without sequelae.

**Fig. 1:**
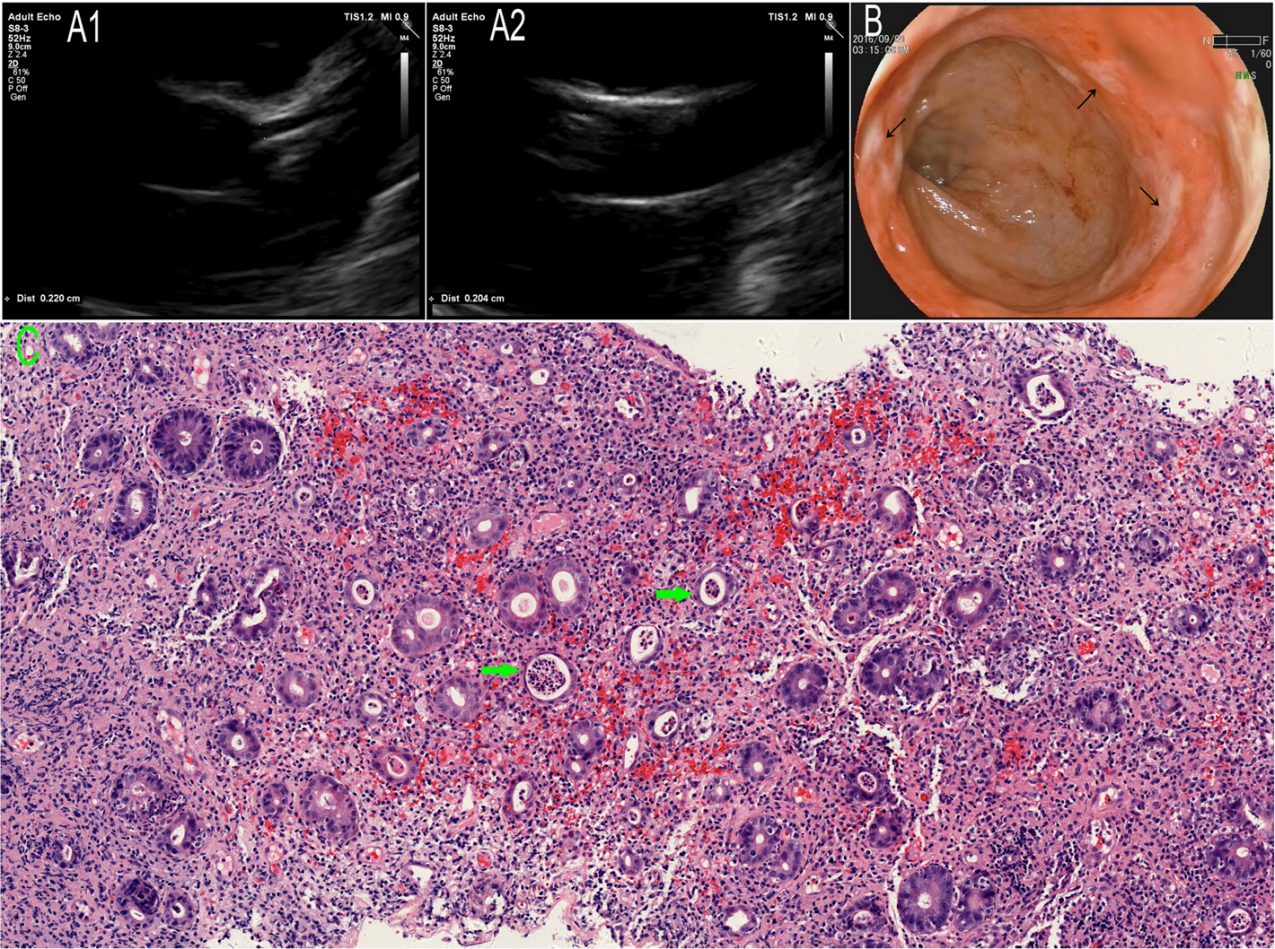
**A**, Echocardiography shows no dilatation of left coronary artery (A1) and right coronary artery (A2). **B**, Colonoscopy results showing multiple superficial active ulcers covered with thick “white moss” in rectum. **C**, Histologic examination of biopsy shows prominent neutrophilic infiltration within the lamina propria along with hemorrhage and crypt abscess formation (green arrow), indicating focal active colitis (hematoxylin-eosin, magnification × 100)

Only 5 cases of KD with gastrointestinal hemorrhage have been previously documented in the English literature (Table 1). Two cases with upper gastrointestinal bleeding occurred before the use of aspirin [1, 2]. Three cases of upper gastrointestinal hemorrhage complicating high-dose aspirin therapy might be iatrogenic [3, 4]. Adverse effects of nonsteroidal anti-inflammatory drugs (NSAIDs) on the lower gastrointestinal tract are also common; however, symptomatic NSAID-induced colitis is rare. Usually, histologically, there is no predominant cell type producing infiltration in colitis associated with NSAIDs [5]. Although the duration of exposure to NSAIDs before the development of symptoms of colitis is variable; most of the patients with NSAID-induced colitis have been taking these drugs for more than 6 months [6]. Our patient developed lower gastrointestinal bleeding on day 3 of aspirin, due to active rectal ulcers. Histological examination of a colonoscopic biopsy specimen revealed prominent neutrophilic infiltration and crypt abscess formation, indicating focal active colitis. In light of clinical details and histological features, we suspect that aspirin may not be the primary cause of rectal ulcers in our patient. Vasculitis of the submucosal artery in the rectum together with the local pathogens might play an important role. Undoubtedly, we can not rule out that high-dose aspirin may play a promoting role. As a matter of fact, the role of high-dose aspirin in KD remains controversial. Kuo et al. [7] demonstrated that high-dose aspirin in KD did not confer any benefit with regards to inflammation and it did not appear to improve treatment outcomes. A recent meta-analysis revealed that low-dose aspirin (3–5 mg/kg/d) may be as effective as the use of high-dose aspirin (> 30 mg/kg/d) for the initial treatment of KD [8]. In summary, pediatricians should be aware of the risk of gastrointestinal bleeding in KD children, especially in those receiving high-dose aspirin.

**Table 1:** Clinical characteristics of the reported patients with gastrointestinal hemorrhage in Kawasaki disease

Ref	First author	Age/sex	Clinical onset	Details of gastrointestinal bleeding	Blood transfusion	Endoscopy	Surgery
1	Zulian	20 m/M	Fever	Hematemesis on day 7 of illness without aspirin	Yes	Showed diffuse hemorrhagic duodenitis	None
2	Singh	4.5 y/M	Nausea, vomiting, diarrhea and fever	Hemorrhagic shock with hematemesis and hematochezia at 2 months after onset (without aspirin)	Yes	Failed because of the massive bleeding	Revaeled a 1.5 cm duodenal ulcer with active bleeding
3	Matsubara	2 y/M	Fever	Hematemesis on day 19 of illness (day 13 of aspirin)	Yes	Revealed a 2 cm duodenal ulcer	None
		4 y/F	Fever	Melanotic stools followed by emesis of blood on day 31 of illness (day 26 of aspirin)	Yes	None	None
4	Chang	5 y/M	Fever, cervical lymphadenitis	Tarry stool on day 6 of illness; Massive gastrointestinal bleeding on day 14 of illness (day 5 of aspirin)	Yes	NA	NA

*Ref* Reference, *y* Year, *m* Month, *M* Male, *F* Female, *NA* Not available

## Data Availability

The datasets used and/or analyzed during the current study are available from the corresponding author on reasonable request.

## Abbreviations

IVIG: Intravenous immunoglobulin
KD: Kawasaki disease
